# Happiness among South African private sector physiotherapists

**DOI:** 10.4102/sajp.v74i1.421

**Published:** 2018-03-28

**Authors:** Michael Elliot, Margaret Cullen, Andre P. Calitz

**Affiliations:** 1 Business School, Nelson Mandela University, South Africa; 2 Department of Computing Sciences, Nelson Mandela University, South Africa

## Abstract

**Background:**

Happiness of people can affect their daily functioning and work performance. There is limited research assessing the happiness levels of various disciplines within the health care industry. This article is the first attempt to evaluate the happiness levels of private sector physiotherapists in South Africa.

**Objectives:**

Research in happiness and physiotherapy studies are two research areas that are not associated with one another in a global perspective. The objective of this study was to assess the happiness levels of private sector physiotherapists in South Africa.

**Methods:**

A hypothesised model was statistically tested using a quantitative questionnaire, which was completed online. The target population of this study were all private sector physiotherapists who are members of the South African Society of Physiotherapy. A total of 395 respondents participated in the study.

**Results:**

This study confirmed that factors such as *influence, social relations, life balance, optimism, work* and *leisure* are all positively associated with the happiness levels of private sector physiotherapists in South Africa. These variables are recommended as key focus areas for physiotherapy practice owners to address, in order to positively affect the happiness levels of all people in their workplace.

**Conclusion:**

The study concludes the following: if happiness becomes a priority, then owners of physiotherapy practices need to generate a workforce who are more productive, demonstrate greater collaboration with colleagues and patients, are more positively energised, are less absent and are more loyal to the practice.

**Clinical Implications:**

The contribution of this study is that it highlights the importance of managing staff in private physiotherapy practices in a holistic manner.

## Introduction

Researchers of positive psychology have progressively increased the awareness of individual happiness studies and their benefits to society (Flynn & MacLeod 2015). Although researchers and lay people often define happiness as life satisfaction or a sense of well-being, studies also define happiness as positive subjective experiences (Delle Fave et al. 2016; Joshanloo & Weijers 2014; Scorsolini-Comin & Dos Santos 2010; Wren-Lewis 2014). However, despite the variance in happiness definitions, research confirms that an increase in individual happiness is advantageous to the individual and also enables societies to function better, thereby supporting the notion of incorporating aspects of happiness when formulating economic policy (Flynn & MacLeod 2015; Guzi & De Pedraza García 2015; Powdthavee 2007).

The multiple dimensions of happiness mentioned above and their cross-country differences or similarities may arguably be because of the variance in cultural dimensions and their collective influence on the happiness of individuals and societies, such as the Hofstede’s dimensions (Delle Fave et al. 2016; Ye, Ng & Lian 2015). Hofstede’s dimensions are measurements of a country’s ‘cultural style’ that were constructed based on the basic problems that all societies could be faced with (Harvey 2011; Minkov & Hofstede 2011).

Theories such as the *comparison theory* and Maslow’s *needs theory* support the fact that culture is linked to happiness or subjective well-being (Diener & Lucas 2000; Schyns 1998; Ye et al. 2015). The comparison theory relates to the degree to which happiness is dependent on the comparisons between the standards of quality of life and the perceived life circumstances, whereas Maslow’s needs theory states that as more needs of an individual are met, the happier the individual will be (Aydin 2012; Diener & Lucas 2000; Schyns 1998; Ye et al. 2015).

A study undertaken to explain the impact of cultural variables on happiness across different countries confirms that Hofstede’s power distance dimension correlates negatively with happiness and that raising gender equality may also improve a country’s happiness measurement (Ye et al. 2015). Furthermore, Ye et al. (2015) state that individualist cultured countries such as European countries and the USA countries are happier or have higher levels of subjective well-being than people in a collectivist culture such as East Asian countries. This is confirmed by studies such as that conducted by Stearns (2012) who states that East Asian cultures have lower happiness expectations than what European and American cultures are accustomed to (Chiu et al. 2011; Stearns 2012; Ye et al. 2015). Culture is therefore an important factor to consider when measuring happiness across countries, and the measurement of happiness across countries is in itself very important (Diener 2000; Diener, Diener & Diener 1995; Heukamp & Ariño 2011; Inglehart & Klingemann 2000; Kenny 1999; Ye et al. 2015).

Although happiness predominantly features in the research disciplines of philosophy, religion and psychology, it has subsequently become a focus in the fields of sociology, economics and neurology. As a result, it has been rather influential in the formulation and publication of public policy (Aydin 2012; Frey et al. 2008; Okulicz-Kozaryn 2016). Economists have focused on happiness studies because of their relevance and the effects of institutional conditions such as quality of governance and the size of social capital on individual well-being (Frey & Stutzer 2002). Frey and Stutzer (2002) further mention that such studies assist economists to understand the formation of well-being and the prediction of societies and individuals’ future utilities. The concept of utility refers to the choices people make in relation to tangible goods, which influence the supply and demand theory of economics and as a result measures of happiness serve as proxies for utility (Frey & Stutzer 2002).

The literature confirms that although, in previous decades, wealthier people were generally happier than poorer people in the same country, developed countries are not necessarily happier than developing countries and in addition concluded that the increase in income over time failed to increase happiness levels of people (Easterlin 2003; Fox 2003; Robinson, Kennedy & Harmon 2012).

Happiness studies have thus been of great importance in the business sector. Pryce-Jones and Lindsay (2014:51) define happiness at work as the ‘mind-set which enables action to maximise performance and achieve potential’. Happiness at work may also be described as ‘the worker’s experience of safety and healthiness of work, good leadership, competence, change management, support at work and how meaningful the employee finds the work’ (Anttonen & Vainio 2010:1246; Utriainen et al. 2011).

This is particularly relevant in the service sector industry, where it has been established that a causal link exists between happy customers and greater profits (Chun & Davies 2009). However, Chun and Davies (2009) state in an article in the Harvard Business Review that the same factors that increase customer satisfaction and in turn generate higher profits, actually have a negative correlation on the happiness of employees.

Hence, because increasing both the customer satisfaction levels and employee happiness is crucial and beneficial to business, it is suggested to link the two by engaging employees with reasons and ways to please customers and thereafter acknowledge and reward appropriate staff behaviour. This in turn assists employees to have a sense of achieving their full potential (Chun & Davies 2009; Pryce-Jones & Lindsay 2014).

The business model of many private sector physiotherapy practices is under pressure because of changes in the health care market forces, causing the costs of running private physiotherapy practices to escalate, whilst the remuneration for such physiotherapy services is diminishing (Ijntema, Mollema & Duits 2016; Neidhardt 2009). The need to modify the business models of physiotherapy practices is essential for practice owners to ensure that they maintain and improve their market share in this particular service industry (Neidhardt 2009). However, a general problem is that physiotherapy practice owners are not equipped to understand where and how these changes should be made, and in addition, information to support such decisions is not readily available (Ijntema et al. 2016; Neidhardt 2009).

In addition to the business model, many private sector physiotherapy practices have started up without the consideration of a business plan, which indicates that factors such as staff recruitment and retention strategies, competition and market segments and business strategies have been either neglected or poorly planned prior to start up because of the lack of business acumen gained from their tertiary education (Desveaux et al. 2012; Wassinger & Baxter 2011). This could therefore lead to mismanagement of business processes and poor leadership attributes that may negatively influence the happiness levels of private sector physiotherapy staff.

A pioneering Canadian study established that the physiotherapy workforce identified a critical need for business acumen (Desveaux et al. 2012). Thirty-eight per cent of the physiotherapists surveyed worked in the private sector and exceeded the expected perceived importance of business acumen in their workplace setting, whereas the remaining 62% of public employed physiotherapists demonstrated a decline in the expected perceived importance of acquiring and implementation of business acumen (Desveaux et al. 2012). The contrast highlighted by Desveaux et al. (2012) relates to the general problem that physiotherapists are not equipped with sufficient business acumen to understand the business aspects of their practice environment and the important relation of business acumen to their practice and management of their staff.

It is therefore evident that physiotherapy practice owners need to identify and analyse aspects of their practices that require attention, improvement and managerial execution of solutions for the problems identified (Neidhardt 2009). This article identifies that happiness levels of private sector physiotherapists in South Africa have not been analysed and that physiotherapy practice owners have limited business knowledge to improve the happiness levels of their staff and thereby improve the success of their practices. This relates to the notable problems in the health care industry across professions, such as staff retention, where physiotherapy practice owners fail to recognise their employees as internal customers and strategic key partners (Ijntema et al. 2016).

There is insufficient information available to know whether private sector physiotherapists in South Africa are happy. This investigation may enable physiotherapy practice owners to implement evidence-based retention and recruitment strategies for high performing physiotherapy staff and in turn generate a workforce that is more productive, demonstrates greater collaboration with staff and patients, produces happier patients, is more positively energised, is less absent and is more loyal to the practice (Kjerulf 2013; Job Satisfaction Index 2015; Pryce-Jones & Lindsay 2014).

Thus far, few studies have been conducted to assess the quality of life, life satisfaction or subjective well-being of physiotherapists in the global health care sector, and to the authors’ knowledge, there has been no attempt to measure these variables in the South African private physiotherapy sector. However, studies of happiness have been progressively conducted internationally and among the local nursing sector of southern Africa. Studies relating to happiness levels of public sector nursing staff in southern Africa indicated that nursing staff are unhappy with their working environment and dissatisfied with the management skills of their supervisors and that the organisational cultures are not conducive to generate high levels of job satisfaction or happiness at work (Pietersen 2005). Therefore, further studies of happiness in the international and national health care sector are necessary across the medical professionals such as nurses, physiotherapists and medical officers, to determine their levels of satisfaction and happiness at work. It is noted that happiness would benefit from multifaceted methodological and theoretical perspectives, such as ethnographic approaches, to understand how health care professionals still experience happiness despite difficult and stressful conditions at work (Einarsdóttir 2012).

The private physiotherapy sector is representative of a business operating within the service industry, as the product consumed is the service rendered by the physiotherapist. Furthermore, these private sector physiotherapists in South Africa form a diverse cultured workforce such as in Australia (Adams et al. 2014) and require studies to support the management of practice owners to thereby ensure that their staff maintain favourable happiness levels at work and in turn generate happy staff and patients.

The literature on happiness uses various measurement instruments in their quest to determine the level of happiness of a specific population and thereby contribute to happiness research, which is gradually becoming a field of primary importance (Dambrun et al. 2012). Dambrun et al. (2012) present the development of two measurement scales of happiness, which intimately link to a preceding theoretical model named the self-centeredness or selfless happiness model presented by Dambrun and Ricard (2011). The two scales of measurement by Dambrun et al. (2012) are the Subjective Fluctuating Happiness Scale (SFHS) and the Subjective Authentic-Durable Happiness Scale (SA-DHS), which are characterised by high internal consistency, a logical factorial structure and stability over time.

A more widely used measuring instrument of happiness is the Oxford Happiness Inventory (OHI), which is a broad measure of personal happiness developed in the late 1980s (Bekhet et al. 2008). This measurement instrument has been used cross-culturally on a worldwide platform and forms the foundation of the Chinese Happiness Inventory (Bekhet et al. 2008). The OHI has been improved to the Oxford Happiness Questionnaire (OHQ) containing similar items to the OHI and is presented on a uniform Likert scale, which is less susceptible to questionnaire and respondent bias (Hills & Argyle 2002). Moreover, a short-form version of the OHQ was devised for use when space and time are limited (Hills & Argyle 2002). The correlation between the results of the full scale and short-form scale is greater than 0.90 and deemed highly significant, *p* < 0.001 (Hills & Argyle 2002).

A recent study conducted among the undergraduate students at the Nipissing University in Canada used the OHQ as a measuring instrument to explore the relationship between happiness and six domains, namely academic success, financial security, familial support, living environment, self-image and social relations (Flynn & MacLeod 2015). However, to date, no studies have used the OHQ or any other valid measure of happiness to determine the level of happiness of physiotherapists in the private sector of South Africa.

**FIGURE 1:**
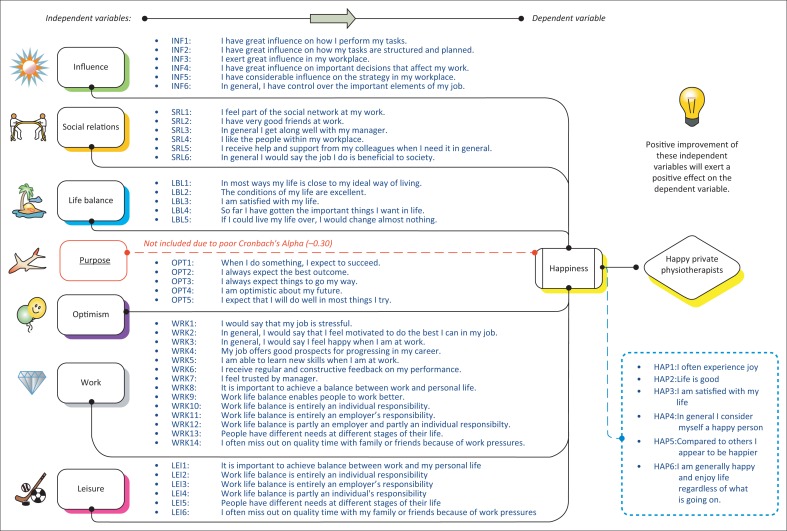
Conceptual model.

Previous studies relating to happiness were used to identify the independent variables for this study in combination with OHQ, which is a proven reliable research instrument. The following independent variables were selected and investigated in this study: *influence, social relations, purpose, life balance, optimism, work* and *leisure.* The dependent variable was *happiness.* The conceptual model created for this study and related survey items are presented in Figure 1.

At present, there is no literature addressing the happiness levels of private sector physiotherapists in South Africa. Therefore, this study may establish a foundation for future research to further investigate and contribute to the body of knowledge with regard to happiness in the health care sector and in addition empower physiotherapy practice owners to increase the happiness levels of their staff, thereby gaining the confirmed benefits that increased employee happiness produces. In addition, this study may provide insight as to what the private sector physiotherapy workforce of South Africa perceive as important in terms of happiness in the workplace.

## Methodology

### Research design

This study followed a positivistic approach and used a quantitative survey strategy to collect data and achieve the primary objective to measure the happiness levels of private sector physiotherapists in South Africa. Quantitative analysis methods are associated with positivistic research, as variables are believed to be measurable (Collis & Hussey 2013). Hence, the primary objective of the study was designed to investigate the causal relationship between the dependent variable *happiness* and its independent variables *influence, social relations, life balance, optimism, work, leisure* and *purpose* (Amaratunga et al. 2002; Collis & Hussey 2013; Creswell 2014).

### Survey design

In order to explore the causal relationship between happiness and its independent variables, a survey was carried out on the target population via an online questionnaire, the short-form, adapted version of the OHQ (Hills & Argyle 2002). The dimensions of *influence, social relations, life balance, optimism, work, leisure* and *purpose* and the dependent variable *happiness* (Figure 1) were identified for this study (Amaratunga et al. 2002; Collis & Hussey 2013; Creswell 2014). The operationalisation of the survey items listed in Figure 1 was obtained from the literature and the OHQ. Exploration of the causal relationship would determine the happiness levels of private sector physiotherapists in South Africa. Hence, this sets the frame for the target audience and target population.

The questionnaire consisted of two main divisions following the introduction: the first division (Section A) captured the demographic aspects of the target population, namely the respondents’ age, gender, tenure, geographical location and education. The next division (Section B to Section G) collected data of the independent and dependent variables through a total of 56 five-point Likert scale statements to capture the respondent’s perception of the identified independent variables as well as the dependent variable. The latter division comprised the following information perused in the literature review with regard to the determining factors of happiness and factors influencing happiness in the workplace.

### Hypotheses

With reference to the literature review, it was hypothesised that *influence, social relations, life balance, purpose, optimism, work* and *leisure* would exert a positive influence on *happiness* of private sector physiotherapists in South Africa. The following hypotheses were formulated in alignment with the conceptual model to establish the relationship between the dependent variable and independent variables:

**H0**_**1**_: *Influence* exerts no effect on happiness.

**HA**_**1**_: *Influence* exerts a positive effect on happiness.

**H0**_**2**_: *Social relations* exert no effect on happiness.

**HA**_**2**_: *Social relations* exert a positive effect on happiness

**H0**_**3**_: *Life balance* exerts no effect on happiness.

**HA**_**3**_: *Life balance* exerts a positive effect on happiness.

**H0**_**4**_: *Purpose* exerts no effect on happiness.

**HA**_**4**_: *Purpose* exerts a positive effect on happiness.

**H0**_**5**_: *Optimism* exerts no effect on happiness.

**HA**_**5**_: *Optimism* exerts a positive effect on happiness.

**H0**_**6**_: *Work* exerts no effect on happiness.

**HA**_**6**_: *Work* exerts a positive effect on happiness.

**H0**_**7**_: *Leisure* exerts no effect on happiness.

**HA**_**7**_: *Leisure* exerts a positive effect on happiness.

### Data collection tool

The Nelson Mandela University (NMU) online survey tool was used to administer the online questionnaire and generate descriptive statistics. The quantitative data sets were further analysed with the IBM Statistical Package for the Social Science (SPSS, version 24) to produce additional descriptive and inferential statistics. Descriptive statistics, Cronbach’s alpha coefficients, Pearson’s correlation values and multiple regression analysis results are reported in this article.

### Reliability and validity

Cronbach’s alpha coefficients were determined to measure the reliability of the variables used in the questionnaire to survey the targeted respondents. Analyses of the data revealed that the overall Cronbach’s alpha coefficient (0.88) was acceptable for the 56 items related to the independent variables identified and that the measuring instruments demonstrated strong internal consistencies. All variables (Table 1), except *purpose*, demonstrated a Cronbach’s alpha above 0.70, which indicates acceptable, internal consistency and reliability of the research questionnaire (Jack & Clarke 1998; OECD 2013; Rattray & Jones 2007). *Purpose* was, however, removed and was not used for interpretation because of the negative average covariance between the question statements.

**TABLE 1:** Cronbach’s alpha coefficients of happiness questionnaire.

Variables	Cronbach’s alpha
Happiness	0.83
Influence	0.90
Social relations	0.80
Life balance	0.89
Optimism	0.72
Work	0.72
Leisure	0.82
Purpose	−0.30

In order to ensure adequate validity supplementary to the above established reliability, face, construct and content validity were upheld through a thorough literature review and grounding the research questionnaire on previously validated questionnaires and surveys on happiness studies.

### Study population and sample

The surveyed sample population for this study represented the target population of all private sector physiotherapists working in South Africa. The target population comprised private sector physiotherapists, who are currently registered either with the Health Professional Council of South Africa (HPCSA) or the South African Society of Physiotherapy (SASP). More than 90% of the respondents were affiliated with the SASP, and respondents indicated that they were registered with the HPCSA. Furthermore, the target population excluded physiotherapists who are employed in the public sector, as this study focused on physiotherapists owning their own practice or who were employed in a private practice. A total of 1289 respondents viewed the questionnaire, from which 507 (39%) started the questionnaire. Finally, 395 (31%) of the respondents successfully completed the online questionnaire.

### Ethical considerations

Ethical approval was obtained from the NMU Business School Ethics Committee (Nelson Mandela University Business School Ethics No: BS Elliot 2016) and permission to conduct the study from the South African Society for Physiotherapy (SASP).

## Results

The descriptive statistics in Table 2 indicate that the majority of the respondents (*n* = 168, 42.5%) reside in the Gauteng Province of South Africa. The majority fall into the age groups 20–30 and 31–40 years of age (*n* = 249, 63%) and are female (*n* = 347, 88%). Two hundred and seventy-two respondents (69%) were married, 63% (*n* = 249) obtained a 4-year degree, 44% have up to 10 years working experience and two-thirds of the respondents are owners of private physiotherapy practices (66%, *n* = 262). The analysis of variance results is presented in Table 3. The five-point Likert scale values were in the range of 1 (Strongly Disagree) to 5 (Strongly Agree). All variables have mean scores between *agree* and *strongly agree* for the 395 respondents.

**TABLE 2:** Demographic frequency distribution (*n* = 395).

Question	Frequency of responses	Percentage
**Q1.1 In which province do you reside?**		
Gauteng	168	42.5
Western Cape	94	23.8
Northern Cape	5	1.3
Eastern Cape	23	5.8
Free State	26	6.6
North West	6	1.5
Mpumalanga	14	3.5
Limpopo	11	2.8
KwaZulu-Natal	48	12.2
Total	395	100
**Q1.2 What is your age?**		
20–30	104	26.3
31–40	145	36.7
41–50	74	18.7
51–60	52	13.2
61–70	20	5.1
Total	395	100
**Q 1.3 What is your gender?**		
Female	347	87.9
Male	48	12.1
Total	395	100
**Q 1.4 What is your marital status?**		
Single	54	13.7
In a relationship	22	5.6
Living together	23	5.8
Married	272	68.8
Divorced	18	4.6
Widowed	6	1.5
Total	395	100
**Q 1.5 What is your highest level of education?**		
Undergraduate	57	14.4
Four-year degree	249	63.1
Master’s	59	14.9
Doctorate	5	1.3
Other	25	6.3
Total	395	100
**Q 1.6 What is the tenure of your experience as a physiotherapist in the private sector?**		
0–10 years	174	44.0
11–20 years	121	30.6
21–30 years	67	16.9
31–40 years	24	6.2
40+ years	9	2.3
Total	395	100
**Q 1.7 Are you a physiotherapy private practice owner?**		
Yes	262	66.3
No	133	33.7
Total	395	100

**TABLE 3:** Analysis of variance.

Variable	Mean	Standard deviation	*n*
Happiness	3.9692 [3.97]	0.62132	395
Influence	4.0447 [4.05]	0.75399	395
Social relations	3.8639 [3.90]	0.70890	395
Life balance	3.5944 [3.60]	0.82957	395
Optimism	3.8927 [3.90]	0.52416	395
Work	3.9494 [3.95]	0.47380	395
Leisure	4.2422 [4.24]	0.53249	395

Table 4, the Pearson’s correlation coefficients, indicates that *happiness* has a high positive correlation with *life balance* (0.74) and a moderate positive correlation with *work* (0.52), *social relations* (0.42), *optimism* (0.40) and *influence* (0.49). However, the dependent variable illustrates a poor positive correlation with *leisure* (0.30).

**TABLE 4:** Pearson’s correlations with *p*-values (*n* = 395).

Correlations	Variables	HAPP	INF	SRL	LBL	OPT	WRK	LEI
Pearson’s correlation	Happiness (HAPP)	1.000	0.487	0.418	0.741	0.397	0.522	0.301
	Influence (INF)	0.487	1.000	0.438	0.497	0.250	0.576	0.100
	Social relations (SRL)	0.418	0.438	1.000	0.441	0.208	0.604	0.133
	Life balance (LBL)	0.741	0.497	0.441	1.000	0.363	0.571	0.189
	Optimism (OPT)	0.397	0.250	0.208	0.363	1.000	0.306	0.335
	Work (WRK)	0.522	0.576	0.604	0.571	0.306	1.000	0.215
	Leisure (LEI)	0.301	0.100	0.133	0.189	0.335	0.215	1.000
Sig. (1-tailed)	Happiness (HAPP)	-	0.000	0.000	0.000	0.000	0.000	0.000
	Influence (INF)	0.000	-	0.000	0.000	0.000	0.000	0.024
	Social relations (SRL)	0.000	0.000	-	0.000	0.000	0.000	0.004
	Life balance (LBL)	0.000	0.000	0.000	-	0.000	0.000	0.000
	Optimism (OPT)	0.010	0.000	0.000	0.000	-	0.000	0.000
	Work (WRK)	0.000	0.000	0.000	0.000	0.000	-	0.000
	Leisure (LEI)	0.000	0.024	0.004	0.000	0.000	0.000	-

The *p*-value for all the independent and dependent variables is below 0.05 and thus illustrates significant relationships (Collis & Hussey 2013; Wegner 2013).

Furthermore, the results concluded that the independent variables are able to cause positive effects on the dependent variable, *happiness. Happines*s is thus moderately correlated with *influence, social relations, life balance, optimism* and *work* and poorly correlated with *leisure.* The hypotheses were evaluated using a multiple regression analysis (Table 3). The following findings were derived from this study:

### Influence

*Influence* is shown to be moderately correlated with *happiness* (*r* = 0.5, *p* < 0.01), and the statistical results indicate that the majority of the respondents agree they exert an influence in their workplace in terms of the capacity to have an effect on the character, development or behaviour of someone or something, or the effect itself. HO_1_ was rejected and HA_1_ was accepted.

### Social relations

*Social relations* with colleagues is shown to be positively poorly correlated with *happiness* (*r* = 0.4, *p* < 0.01), and the statistical results indicate that the majority of the respondents agree they have strong social relations with their colleagues in terms of how much value they place on the love and support of their colleagues. HO_2_ was rejected and HA_2_ was accepted.

### Life balance

*Life balance* is shown to have a good correlation with *happiness* (*r* = 0.7, *p* < 0.01), and the statistical results thus indicate that the majority of the respondents agree they experience a balanced life, encompassing positive self-reported indications of their well-being. This independent variable demonstrates the highest positive correlation with happiness. HO_3_ was rejected and HA_3_ was accepted.

### Optimism

*Optimism* is shown to be poorly correlated with *happiness* (*r* = 0.4, *p* < 0.05), and the statistical results indicate that the majority of the respondents agree to being optimistic regarding their convictions that things will always turn out well. HO_5_ was rejected and HA_5_ was accepted.

### Work

*Work* is shown to be moderately correlated with *happiness* (*r* = 0.5, *p* < 0.01), and the statistical results indicate that the majority of the respondents agree with the notion that paid work and personal life should be seen less as competing priorities than as complementary elements of a full life. HO_6_ was rejected and HA_6_ was accepted.

### Leisure

*Leisure* is shown to be poorly correlated with *happiness* (*r* = 0.3, *p* < 0.01), and the statistical results indicate that the majority of the respondents agree they perceive their leisure activities as important and enjoyable. HO_7_ was rejected and HA_7_ was accepted.

As a result, it is therefore established that the independent variables exert a moderate to low positive effect on the happiness levels of private sector physiotherapists in South Africa.

The multiple linear regression analysis R^2^ values indicate that approximately 60% of the variance in private sector physiotherapists’ happiness may be explained by the combined effect of four independent variables, namely *life, leisure, influence* and *optimism* (Table 5).

**TABLE 5:** Multiple regression analysis. Model summary^a^

Model	*R*	*R* square	Adjusted *R* square	Standard error of the estimate	Durbin–Watson
1	0.741^b^	0.549	0.548	0.41791	-
2	0.759^c^	0.576	0.573	0.40582	-
3	0.771^d^	0.594	0.591	0.39749	-
4	0.775^e^	0.601	0.597	0.39464	1.981

a , Dependent variable: *happiness*;

b , predictors: (constant), *life*;

c , predictors: (constant), *life, leisure*;

d , predictors: (constant), *life, leisure, influence*;

e , predictors: (constant), *life, leisure, influence, optimism*.

### Proposed conceptual model

**FIGURE 2:**
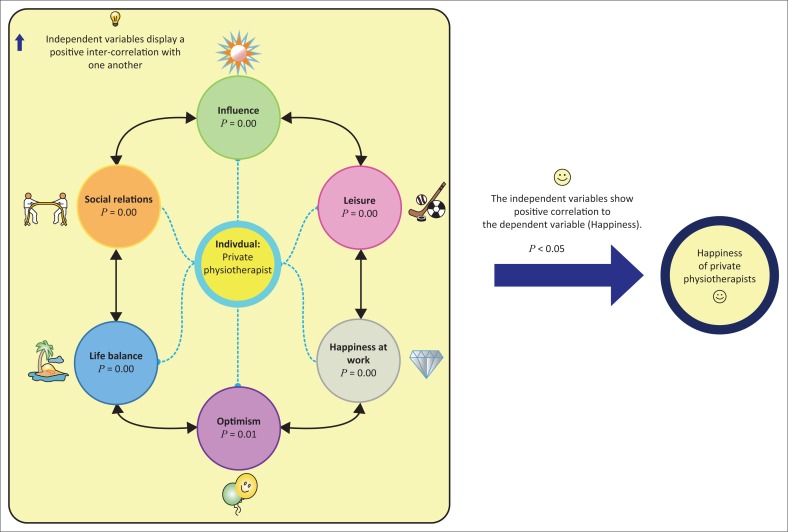
Statistical model for enhancing happiness of private physiotherapists in South Africa.

The statistical analyses verify that *influence, social relations, life balance, optimism* and *work*, which were the chosen independent variables, are moderately associated with the *happiness* of private sector physiotherapists in South Africa. The statistical analyses also illustrates that there is a low to medium degree of positive inter-correlation between the independent variables, as measured by the Pearson’s coefficient and the multiple regression analysis. In light of this low to medium strength of positive inter-correlation between the independent variables and the conjunction of significant positive effect these independent variables have on happiness, the following model in Figure 2 is proposed to enhance the happiness of private sector physiotherapists in South Africa.

## Discussion

This study is the first attempt to determine the happiness levels of physiotherapists in South Africa and thus provides deeper insight into what the private sector physiotherapy workforce of South Africa perceives as important in terms of happiness at the workplace. The empirical evidence above and the integrated proposed model may enable physiotherapy practice owners to focus on their workforce and their well-being. They are more likely to generate and share new ideas and create greater cooperation between colleagues and with patients. They could become more energised, more loyal to their practices and possibly more productive in accordance with the established benefits of increased happiness, highlighted in the literature review (Job Satisfaction Index 2015; Kjerulf 2013; Pryce-Jones & Lindsay 2014).

### Limitations

This study was conducted in one country and a concern was that of sampling bias, as most of the respondents were private sector physiotherapy practice owners. The measuring instrument could be more refined to further investigate whether they are the only service provider of the practice or how many staff they have employed as well as the nature of their employment. Strategies need to be investigated to obtain a better response rate in future studies. Lastly, the measuring instrument did not measure whether those respondents who were employed were permanent or temporary physiotherapists.

### Future research

Opportunities for further research are identified as follows:

- Compare the happiness values of permanent staff against temporary staff or compare physiotherapists who employ themselves to be the sole service provider versus those who choose to employ other physiotherapists and work in a team environment.
- Conduct a longitudinal study, which could provide a longer time frame to adequately refine the measuring instrument and include public sector employed physiotherapists into the sample. The comparison of happiness levels between private sector physiotherapists and public sector employed physiotherapists would enable greater and substantial contributions to the health care sector regarding allied health care professions such as physiotherapy. The managerial implications may differ between these two physiotherapy groups and provide further insight for government to generate a workforce that stimulates the established benefits of happiness in the workplace.
- Distinguish between physiotherapy groups – physiotherapists work in a multitude of disciplines such as sports physiotherapy, outpatient physiotherapy (traditional stand-alone practices), inpatient physiotherapy (operating out of hospitals), a combination of inpatient and outpatient physiotherapy, neurological rehabilitation physiotherapy centres; the variance of happiness levels across these sectors would prove as substantial contributions to the field investigated.
- Although previous studies have conducted happiness studies in the nursing sector within southern Africa, further research is warranted in the physiotherapy profession as well as across the various disciplines in the health care sector. A comparison between the multidisciplinary health care professionals regarding their happiness levels and causal factors will make for a potentially useful longitudinal study.
- Further research could be conducted to determine whether the respondents would change their career choice to that of medicine for example.
- A statistical model was formulated in light of the empirical findings and warrants further testing to verify its credence.

### Recommendations

In order to implement the pioneering findings of this study, the following recommendations are proposed in light of the empirical evidence and literature review in order to improve the happiness levels of private sector physiotherapists in South Africa:

*Influence*: It allows private sector physiotherapists to take control of how they plan their work, thereby improving their self-efficacy as independent practitioners. In agreement with the results, it would not be wise to micromanage private physiotherapists in terms of how their tasks are planned, how they perform their tasks and execute the important elements of their profession.

*Social relations*: It builds on social networks at work as this was ranked the most negatively. This could be because of the fact that physiotherapists spend most of their working day with their patients during private and confidential treatment sessions and as a result have less time to socialise with one another during working hours. Time should be scheduled to build on social relations and develop strong social networks to promote happiness of the staff and in turn foster loyalty and better collaboration between colleagues. Although the majority felt that they received support from their colleagues, 34% of respondents did not feel that they were part of social networks and 37% did not feel that they had good friends at work. Hence, team-building exercises and placing importance on social relations at work from a managerial perspective is important to drive happiness in the workplace (Kjerulf 2013).

*Life balance*: Much emphasis should be placed on this in comparison with the other independent variables of medium and low correlation. It is recommended that physiotherapy practice owners could acquire a coaching toolkit to assist their staff towards achieving contentment. Alternatively, it is recommended that the practice owner make use of a life coach to assist their employees in becoming their best selves. Through the use of a life coach, the practice owner will be able to manage their staff based on their personality profiles and thereby foster great social relations as well.

*Optimism*: Although this variable positively correlates to happiness with low strength, it is recommended to nurture the highly optimistic nature of the staff to combat the moments when staff is not feeling happy. Hence, the highly optimistic nature of private physiotherapists can be used as an opportunity to generate higher life satisfaction levels and consequently generate happier staff.

*Work*: It is recommended to create avenues that staff may progress through their careers as clinicians and provide incentives for furthering their education. Practice owners need to provide regular and constructive feedback regarding the performance of their staff. Pryce-Jones and Lindsay (2014) suggest that business owners assist employees to achieve their full potential. Hence, performance appraisal systems should be agreed upon in order to provide the employee with constructive feedback and enable them to be more productive. Lastly, practice owners need to ensure that their staff are not overworked to the point where their work satisfaction is compromised. It is further recommended that staff is encouraged to brainstorm and create systems to promote work satisfaction of all staff, thereby involving the employees in strategic discussions and interlinking the *influence* proposal.

*Leisure*: It is recommended that the staff actively engage in leisure activities to alleviate any stress, promote their emotional well-being and enhance their social relations. The authors further recommend that physiotherapy practice owners facilitate leisure activities that the staff can engage in as a team to promote social relations with colleagues. This recommendation will interlink with the *social relations* proposal to build social networks within the workforce (Kjerulf 2013). Thus, providing a platform for leisure activities may improve the social networks as leisure activities are felt to improve the respondent’s ability to develop close relations.

## Conclusions

This study has pioneered a way forward for physiotherapy practice owners to adapt their practice model by aligning their intrinsic operations towards enhanced staff happiness and thus more favourable financial bottom lines via the proposed statistical model. Factors of staff retention and recruitment of high performing staff remain a concern for developing countries, such as South Africa, with regard to the increasing shortage of health care staff, often lost because of immigration factors to more developed countries. Hence, this study contributes to enable physiotherapy practice owners to adapt their business model to counter the changes in the health care market forces. They can thus understand the driving forces behind their organisational culture and thereby develop appropriate retention and attraction strategies and consequently foster happier workforces that generate positive effects on their practice bottom line.
